# Multiphase flow detection with photonic crystals and deep learning

**DOI:** 10.1038/s41467-022-28174-2

**Published:** 2022-01-28

**Authors:** Lang Feng, Stefan Natu, Victoria Som de Cerff Edmonds, John J. Valenza

**Affiliations:** 1 Corporate Strategic Research, ExxonMobil Research and Engineering, 1545 Route 22 East, Annandale, NJ 08801 USA; 2 Research and Engineering IT, ExxonMobil Technical Computing Company, 1545 Route 22 East, Annandale, NJ 08801 USA; 3 Present Address: Amazon Alexa, 7W 34th St, New York, NY 10001 USA

**Keywords:** Applied physics, Characterization and analytical techniques

## Abstract

Multiphase flows are ubiquitous in industrial settings. It is often necessary to characterize these fluid mixtures in support of process optimization. Unfortunately, existing commercial technologies often fail to provide frequent, accurate, and cost-efficient data necessary to enable process optimization. Here we show a new physics-based concept and testing with lab and field prototypes leveraging photonic crystals for real-time characterization of multiphase flows. In particular, low power (~1 mW) microwave transmission through photonic crystals filled with fluid mixtures may be interrogated by deep learning analysis techniques to provide a fast and accurate characterization of phase fraction and flow morphology. Moreover when these flow characteristics are known, the flow rate is accurately inferred from the differential pressure necessary for the flow to pass through the photonic crystal. This insight provides a basis to develop a unique class of inexpensive, accurate, and convenient techniques to characterize multiphase flows.

## Abstract (web summary)

Photonic crystal (PC)-based sensing is an attractive approach for achieving accurate environmental sensing applications due to its band structure. Here, the authors utilize microwave transmission through PCs and deep learning physics-based data analytics to characterize flowing fluid mixtures.

## Introduction

Photonic crystals^1–8^ (PC) consist of perfect spatial arrays of dielectric property contrast that act to govern the transmission of light or electromagnetic radiation, and these structures permit specific modes of wave propagation. Thus, a frequency space band structure arises that consists of stop bands that outline forbidden modes. As a result of this behavior, PCs are amenable to a plethora of environmental sensing applications that relate changes in the photonic band structure to variations in system specific quantities, like concentration or species detection^9–13^.

Multiphase flow measurements (MPFM) consist of a collection of technologies to infer flow characteristics such as, phase fractions^14–18^, flow rates^19–22^, and flow morphology. Conventional techniques to determine these flow characteristics are cumbersome to implement, prone to fouling and error, and require regular calibration^15^. This includes microwave-based phase fraction measurements which either do not sample the entire flow cross-section, or require a support structure or an alternative low-frequency RF band to allow for energy transmission through fluid mixtures with a high water cut^15^.

In this work we investigate the utility of exploiting microwave transmission through PCs for characterizing phase fraction, flow rates, and flow morphology. Unlike previous PC sensing applications which concern subtle changes to the spatially uniform dielectric contrast, our approach focuses on the effects of significantly varying the PC dielectric contrast through global phase substitution. In other words, we seek to determine whether or not it is possible to infer flow characteristics from the spatial distribution of dielectric contrast in a PC filled with the fluid mixture of interest. One means to accomplish this would be to invert microwave transmission data for the spatiotemporal dielectric constant in the structure; However, this approach requires significant computational and financial investment, and ultimately precludes real-time characterization of flow characteristics. Therefore, we also investigate the viability of using Deep learning physics-based data analytics^23^ to interrogate microwave transmission data in support of rapid, easy to deploy, and relatively inexpensive MPFM.

## Results

### Microwave transmission measurement of photonic crystal filled with fluids

**Fig. 1:**
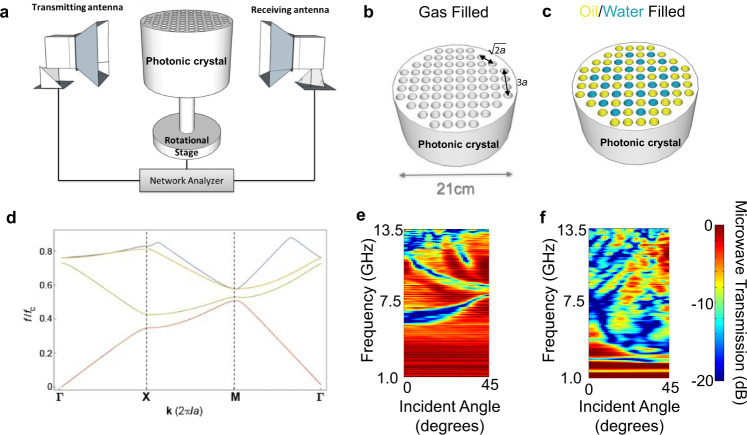
Concept of utilizing photonic crystal to detect multiphase materials. **a** Lab-scale experimental configuration with the transmitting and receiving antenna on opposite sides of the photonic crystal (PC). Both antennas are connected to a network analyzer (see methods). The PC is placed on top of a plastic rod that is connected to a rotational stage. **b** An illustration of the photonic crystal with multitude of holes arranged in a square lattice configuration; In this case the holes are filled with air. Here $a$ is the lattice constant. **c** An illustration of the same photonic crystal structure with a proportion of the holes filled with water (blue) and the remaining holes filled with oil (yellow). **d** Computed photonic band structure of the square lattice (first four eigenmodes are shown, sequentially as red, green, yellow and blue curves). Here **k** is the wave vector and $a$ is the lattice constant. The symmetry points in the first Brillouin zone are shown on the *x*-axis, (**Γ**, **X**, and **M**) and the y-axis is the frequency $f$ normalized by the characteristic frequency ${f}_{c}$. **e**, **f** Color contour plots of the microwave transmission measurements through the structures shown in **b** and **c**, respectively. The *x*-axis is incident angle, with zero defined as the **X** symmetry point in **d** or equivalently vertical direction of **b**. Increasing incident angles indicates rotating in clock-wise direction. The *y*-axis is microwave frequency. The color in **e** and **f** corresponds to the microwave transmission coefficient as indicated in the accompanying scale bar.

We start with a laboratory-scale measurement of microwave transmission through a photonic crystal. The experimental setup is similar to that previously described in the literature^3,5,7,8^. As shown in Fig. 1a, a two-dimensional cylindrical photonic crystal (PC) is mounted on top of a rotational stage between two microwave ridged horn antennas with the central axis of the fins aligned. One antenna irradiates the PC with microwave energy in the TE mode, while the other antenna receives the transmitted TE mode microwaves. Both antennas are connected to a network analyzer (see Methods for more details). As illustrated in Fig. 1b, the PC used in the experiments is made of a cylindrical polyethylene block with a diameter of 20 cm and a height of 15 cm. It contains a square lattice of 1.5 cm diameter holes with a lattice constant of *a* = 2.0 cm. The holes are machined to a depth of 14 cm, leaving a 1 cm thick solid bottom that allows us to fill the holes with liquid. An example of a liquid filled PC is shown in Fig. 1c.

When the microwave energy is imparted on the air-filled (Fig. 1b) PC it is met with a periodic array of binary dielectric contrast. Therefore, photonic band structure governs the transmission of microwave energy over the frequency range 1 GHz to 13.5 GHz employed in our experiments. For an infinitely large PC like that described above, with relative dielectric permittivities of 2.3 and 1.0 for polyethylene and air, respectively, the ideal photonic band structure can be determined by solving Maxwell’s equation^1^. The first four eigenmodes are shown in Fig. 1d, where the frequency is normalized by the characteristic frequency (${f}_{c}=c/a$ where *c* is the speed of light) for the lattice. In the plot, **Γ**, **X**, and **M** indicate the wave vectors that correspond to the symmetry points in the first Brilloun zone^24^. As previously reported^5,7^, we present the angle and frequency-dependent microwave transmission data as a color contour plot like that shown in Fig. 1e (see Methods for more details). Stop bands, corresponding to low transmission, are indicated by green-to-blue color. The contour plot is a direct measurement of the photonic band structure between the **X** and **M** symmetry points (Fig. 1d)^5,7^. It is worth noting, that the band structure shown in (Fig. 1d, e) is essentially the fingerprint of the crystal and thus it may be exploited to calibrate the system response. The structure of the contour plot (Fig. 1f) changes drastically when the holes of the PC are randomly filled with water (blue) or oil (yellow) as indicated in Fig. 1c. The variation in angle and frequency-dependent microwave transmission observed between Fig. 1e, f is due to the different spatial distribution of complex-valued dielectric constants when oil or water is randomly substituted for air. This phase substitution introduces two randomly distributed dielectric contrasts which results in additional scattering and attenuation on top of the conventional photonic physics. While the information in Fig. 1f seems chaotic relative to that shown in Fig. 1e, it is necessarily true that the spatial distribution of complex-valued dielectric constant is encoded in this image. While it is time consuming and computationally expensive to invert this image for the phase distribution in the PC, we investigate the use of a supervised machine learning analysis to predict the phase fractions (oil/water) and macroscopic distribution in the PC. This work is intended to provide a basis for a real-time inference of the phase fraction and flow morphology of multiphase flows. The remainder of the article is organized in the following manner: we start with static laboratory and numerical experiments, then we establish a machine learning protocol to accurately predict phase fraction, flow morphology, and assess the feasibility of field-test prototype PC. Finally, we test the robustness of the collective approach in dynamic experiments at a pilot-scale flow loop.

### Experiments and simulations on multiphase systems

**Fig. 2:**
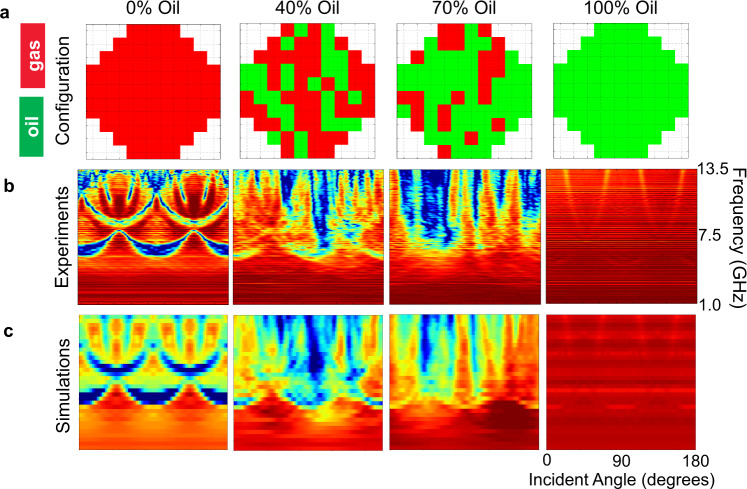
Experimental and simulation contour plots at different oil/gas fractions. **a** Examples of the random configurations of oil-filled holes in the photonic crystal block, with filling fractions (left to right): 0%, 40%, 70% to 100%. The remaining holes are filled with air. Green pixels represent oil-filled holes and red pixels represent air-filled holes. **b** Color contour plots from microwave transmission measurement on photonic crystal with oil-filling pattern corresponding to the configurations in **a**. The contour plots are shown for the range of incident angle 0–180° (*x*-axis) with a step size of 1°, and there are 201 discrete measurement frequencies along the y-axis from 1.0 to 13.5 GHz. The microwave transmission coefficient color scale is the same as that shown in Fig. 1. **c** Color contour plots from the forward model COMSOL simulation of microwave transmission on photonic crystal with oil-filling pattern corresponding to the configurations in **a**. The contour plots are shown for the range of incident angle 0–180° on the x-axis with a step size of 2°, and there are 33 discrete measurement frequencies along the *y*-axis over the range 1.0–13.5 GHz. The microwave transmission coefficient color scale is the same as that shown in Fig. 1.

The accuracy of supervised machine learning data analytics depends almost entirely on providing a training dataset that is representative of the relevant spectrum of realizations encountered in practice. Similarly the resolution of the data analytics prediction is dependent on the sampling frequency over the relevant spectrum of parameter space. In the context of the contour plots discussed previously (Fig. 1e, f), while they represent a considerable amount of information (200 frequencies × 45 angles), it is necessary to obtain a large number of these images corresponding to the full spectrum of oil/water phase fractions constrained by the relevant flow morphologies^25–27^ in order to accurately infer these flow characteristics with a machine learning model. To test the viability of this approach, we performed 100 static experiments in the lab, by completely filling individual holes with a synthetic polyalphaolefin (PAO) oil, where the fraction of filled holes spanned 0% to 100% with a step size of 10% (e.g., 10%, 20%, 30%, etc.). For each experiment a set of holes were selected using a random number generator in Matlab and then filled with oil. The configurations of colored pixels in Fig. 2a provide examples of filling configuration in the PC block, for filling fractions of 0, 40, 70, and 100% oil (green pixels) while the remaining holes are filled with air (red pixels). The patterns corresponding to 40% and 70% filling fractions exhibit the random nature of the oil-filled holes. For each filling fraction other than 0% and 100%, the experiments are performed several times to test different realizations of oil-filled holes at a single filling fraction.

Figure 2b shows four examples of the measured microwave transmission for the oil-filling configurations shown immediately above in Fig. 2a. The contour plots contain 180 equally spaced angles in the range of 0–180 degrees, and 200 equally spaced frequencies in the range 1.0–13.5 GHz. Although the contour plot for 0% oil filling has a well-defined symmetry due to the near-perfect photonic band structure (e.g., as compared to Fig. 1d**X**-**M**), additional data across a broader range of angles could be useful for inferring additional information on material properties, or the spatial phase distributions in the PC as in Fig. 2b (additional experimental data from 0 to 360 degrees can be found in Supplementary Figs. 2, 3 and 4). The nearly isotropic high transmission contour plot at 100% oil fraction is due to a very low dielectric contrast between the polyethylene PC structure (*ε*_*r*_ ~2.3) and the PAO oil-filled holes (*ε*_*r*_~2.1). To complement the lab experiments, we performed finite element COMSOL 2D simulation (see Methods for more details) to generate simulated microwave transmission contour plots for all the oil-filling configurations tested experimentally. Four examples of the simulated color contour plots are shown in Fig. 2c for the same oil-filling configurations considered heretofore. The numerical results are generally consistent with the transmission measurements, exhibiting only minor discrepancies associated with the finite length of PC structure due to the fact that we had to estimate the beam aperture, and the frequency-dependent transfer function of the antennas. The physical experiments are time consuming and it is prohibitively expensive to parallelize our lab effort (Fig. 2b). On the other hand it is common practice to parallelize numerical experiments consisting of running forward models on a multi-core computing cluster (see Methods for more details). Therefore, the good agreement between the experimental and simulated data enables us to drastically increase the number of datasets available to train the machine learning algorithm. This includes accounting for additional complications encountered in flowing liquid systems such as the potential for filling the holes in the PC with mixtures of the liquid components.

### Predictions of phase fractions with deep neural network

**Fig. 3:**
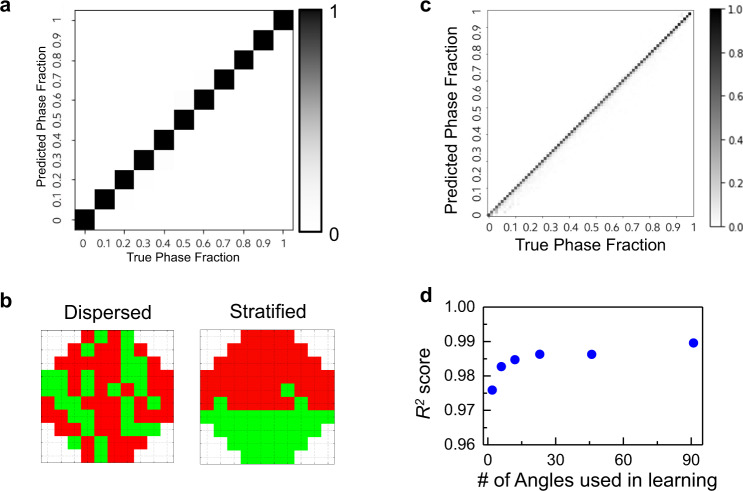
Machine learning predictions with lab-scale data. **a** 2D probability histogram (also known as Confusion Matrix) showing the prediction accuracy for blind test data with the trained neural network model using data collected in the lab-scale experiments (e.g., Fig. 2b). The overall prediction accuracy is 99.5%. The gray-scale color bar indicates the probability of prediction along the *y*-axis given for each true phase fraction (step size 0.1). **b** Examples of a random oil-filling configuration (left) compared to a mostly stratified configuration (right) when 40% of the holes in the photonic crystal are filled with oil. **c** 2D probability histogram showing the prediction accuracy for blind test data with the trained neural network model using simulated transmission data (e.g., Fig. 2c). The overall prediction accuracy is 68% using 8 uniformly distributed frequencies while the ${R}^{2}$ score is 0.990. The gray-scale color bar indicates the probability of prediction along the *y*-axis for each true phase fraction (step size 1.3%). **d** Overall prediction ${R}^{2}$ scores utilizing data from the number of incident angles indicated in the *x*-axis utilizing the machine learning training and testing procedures described in the text. The numbers of incident angles range from 2 to 91, where the angles are uniformly distributed over the range 0–180°.

Next we assess the capability of an open-source machine learning algorithm to analyze the microwave transmission measurements (e.g., as shown in Fig. 2), from lab experimental data to simulated data. Prior to settling on this data analytics approach, we considered various statistical methods^28,29^, effective medium theory^30^, and physics-based computational inversion as potential means to analyze our microwave transmission measurements. These approaches faced many challenges which precluded our ability to predict the flow characteristics with requisite accuracy. We quantify the accuracy of the data analytics model prediction using prediction accuracy and *R*^*2*^. In this context, prediction accuracy is the ratio of the number of exactly correct predictions to the total number of data points, and *R*^*2*^ is 1 minus the ratio of variability in the difference between the model predictions and the data, and the variability in the data (see Methods for more detail on prediction accuracy and ${R}^{2}$ score). With these statistics we assessed the accuracy of several linear machine learning algorithms, such as random forests and support vector machine. Like the conventional analytical techniques these analyses failed to yield the accuracy (e.g., a prediction accuracy of over 98% or a ${R}^{2}$ score over 0.95) required to render these techniques competitive with current technology. Next we turned to Feed Forward Neural Network to train, classify and perform blind tests of 100 experimental measurements consisting of varying the fraction of oil-filled holes in the PC in the range 0–100%. The spectra collected for 360 transmission angles equally spaced over the range 0-360° were randomly split into a set of 80/20 non-overlapping training and blind test data, respectively. Each training dataset consists of the transmission coefficient spectra for one or more incident angles. In the example shown in Fig. 3a, one set of training input data includes a single transmission coefficient spectrum at a specific incident angle of an experimental polar intensity plot. The training dataset along with data labels (fractions of oil) are fed into a supervised Machine Learning classifier through Feed Forward Neural Network to build a training model (see Methods for more details). The accuracy of the training model is then tested on the blind test data. The 2D probability histogram (similar to Confusion Matrix; see Methods) in Fig. 3a shows the prediction accuracy for all the test data predicted with the trained neural network model. The overall prediction accuracy is 99.5%. Also it is particularly significant that after training the neural network model, the phase fraction prediction takes milliseconds, thus this approach enables real-time predictions for industrial applications. (See Methods)

While the deep neural network provides fast and accurate predictions on the fly, it is also desirable to preserve the prediction accuracy while reducing the information (e.g., the number of angles and frequencies) utilized in practice. Reducing the number of incident angles and measured frequencies significantly reduces the cost and complexity of implementing this technique in an industrial setting. As previously mentioned, it is prohibitively cumbersome to produce a representative dataset (e.g., due to the requisite number of experiments) that permits us to test the sensitivity of this technology as we pare down the information utilized in the model.

Given the good agreement between the numerical datasets and our experimental measurements shown in Fig. 2c, we can generate a massive dataset consisting of simulated results on the entire spectrum of relevant realizations of phase distributions in order to test the sensitivity of our technology when we withhold information. We generated 1400 numerical datasets characterized by unique randomly distributed oil-filling configurations with filling fractions ranging from 0 to 100% with a step size of 1.3% (76 holes in total with a one-hole increment for oil-filling fraction). As with the experimental measurements, the forward model is run for several different oil-filling configurations characterized by a single oil-filling fraction. In addition to a random distribution of oil, we also simulated results from 1400 additional configurations that are considered as simplified flow morphologies such as “stratified” or density separated (as the one shown in Fig. 3b on the right). The stratified flow morphology is only possible when the fluid conduit is horizontal and the Reynolds number corresponding to the bulk flow velocity is low. Under these circumstances, when phases do not have equivalent density they separate due to the difference in gravitational potential. In contrast, the random oil-filled configurations (Fig. 2a and Fig. 3b-left) are representative of a “dispersed” flow which is experienced when the bulk flow rate is high and different phases are thoroughly mixed due to the accompanying turbulence. Using a protocol similar to that employed to generate Fig. 3a, we make a binary prediction with the simulated datasets to infer the flow morphology (e.g., “dispersed” or “stratified”) with an accuracy beyond 99%. In addition, as shown in Fig. 3c, we can predict the phase fraction of oils in the system at an overall prediction accuracy of 68% with 8 uniformly selected frequencies between 1 and 13.5 GHz, and 91 angles from 0 to 180 agrees (interval of 2 degrees). In this case the prediction accuracy is modest due to the small step size of 1.3%, and the fact that a prediction is only considered accurate if the predicted and true class are equivalent. In contrast the ${R}^{2}$ is 0.990 for the data shown in Fig. 3c, which more accurately quantifies the small variability in the model prediction exhibited by the fact that the gray-scale pixels are largely confined to the unity line nearest neighbors. (See more detail in Methods)

A key metric for enabling this technology for simple and robust field application is whether the prediction accuracy or ${R}^{2}$ remains acceptable while reducing the amount of information utilized in the analysis. From an ease of implementation standpoint it would be particularly useful to reduce the number of incident angles utilized in the measurement. To address this, we performed a sensitivity study with the numerical dataset to reduce the number of incident angles utilized in all aspects of the data analytics model including training the deep neural network and data validation. The overall prediction ${R}^{2}$ score as a function of the number of incident angles utilized while analyzing the data is shown in Fig. 3d utilizing the same eight frequencies as that used to generate Fig. 3c. The numbers of incident angles range from 2 to 91, and the specific angles are selected uniformly from 0 to 180 degrees. Remarkably, we can still achieve a 97.6% prediction ${R}^{2}$ accuracy for oil phase fractions when we only consider the data from two incident angles (0 and 90°).

### Pilot-scale photonic crystal “Flow Conditioner” for sensing dynamic phase fractions

The results of the multitude of COMSOL simulated transmission data and data analytics in Fig. 3b-d provide confidence that it is possible to accurately predict phase fraction and flow morphology with a very small proportion of the available measurement angles and frequencies. Unlike the static lab experiments, the fluid is flowing in most industrial applications. Therefore, we devised a field pilot experiment at a two-phase flow loop to test the measurement accuracy using a small proportion of angles and frequencies. In addition, we can test for effects associated with the liquid flowing through the PC during the transmission measurement. As it is constituted, the PC is very similar to a traditional “flow conditioner”^31,32^ which typically imparts drag on the fluids that pass through in order to transition turbulent flows to the laminar regime. The additional drag necessitates that the fluid pressure drop between the inlet and the outlet of the PC. Therefore with the addition of a differential pressure measurement across the PC we can test the ability to also infer the flow rate during these experiments.

**Fig. 4:**
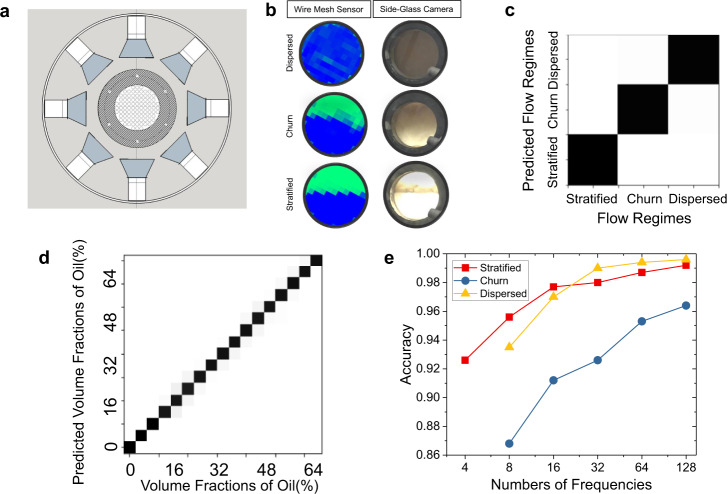
Pilot-scale experiments and the machine learning predictions on fluid phase fractions and flow regimes. **a** Cross-section view of the pilot-scale integrated device with photonic crystal surrounded by four pairs of ridged horn antennas. **b** Example of the various forms of field-test data collected at 44% oil fraction: (left column) wire-mesh sensor and (right column) digital images from side-glass camera. This data are primarily used to confirm the flow regimes which is one of the data labels utilized in the machine learning algorithm. With respect to the wire-mesh data map, green pixels indicate oil and blue pixel indicate water. Top: dispersed flow regime achieved at an average flow rate of about 250 GPM (gallons per minute), Middle: churn flow regime achieved at an average flow rate of about 110 GPM; Bottom: stratified flow regime achieved at an average flow rate of about 60 GPM. **c** The 2D probability histogram of predicted flow regimes (stratified, churn, or dispersed) tested with Convolutional Neural Net and data from the field test. The gray-scale color that indicates the probability of prediction is the same as that shown in Fig. 3a. **d** The 2D probability histogram of predicted oil fraction in the churn flow regime tested with Convolutional Neural Net and data from the field test. The grey-scale color that indicates the probability of prediction is the same as that shown in Fig. 3a. **e** Oil fraction prediction accuracies of the CNN model utilizing data from the number of frequencies indicated on the *x*-axis for the three flow regimes tested in the field. Red, yellow, and blue data points correspond to stratified, churn, and dispersed flow regimes, respectively.

The PC used in field test is made of PEEK (Polyether ether ketone) surrounded by four pairs of transmitting and receiving antennas (e.g., four measurement angles). A cross-section view of the integrated device is shown in Fig. 4a (see Methods and Supplementary Fig. 5 for more details). The PC has a diameter of about 89 mm and a height of about 100 mm, where the diameter is selected so the PC fits in the flow loop pipe (inner diameter of about 102 mm). The holes that make up the square lattice have a diameter of about 5.0 mm with a lattice spacing of 6.67 mm. This square array has a characteristic frequency of ${f}_{c}=45\;{{{{{\mathrm{{GHz}}}}}}}$. PEEK has a relative permittivity of *ε*_*r*_~2.9. The PC is enclosed by a cylindrical wall of PEEK with 3.2 cm thickness to ensure structural integrity under the maximum flow loop pressure of 100 psi. The network analyzer, antennas and multiplexers used in the field testing (see Methods) enabled us to measure at a higher operational frequency range, 4–26 GHz, to explore the relevant signal from the photonic physics for the structure (e.g., Fig. 1d). As shown in Fig. 4a, the transmission measurements are acquired at four different angles: 0, 45, 90, and 145 degrees.

The field test consists of a series of experiments to understand the effects of flowing a mixture of water and oil through the photonic crystal “flow conditioner”. We use IsoparV^TM^ for oil (see Methods) and the tap water supplied at the ExxonMobil Houston Friendswood test facility. We acquired a large amount of data during the field test while the oil fraction was increased from 0 to 64% with a 4% step size. At each oil fraction we modified the flow rate to achieve three distinct flow regimes, stratified, churn, and dispersed. Each dataset includes microwave transmission measurements from four incident angles paired with a cross-section phase map from a research grade wire-mesh sensor (see Methods) and a digital image from a side-glass camera. This data were collected for a total of 2 h at each oil fraction corresponding to roughly 40 min. of data per flow regime. Examples of data from the wire-mesh sensor and the side-glass camera are shown in Fig. 4b for an oil fraction of 44% for all three flow regimes. The wire-mesh data and digital images are primarily used to confirm the consistency of flow regime and fluid distribution over the continuous 2 h testing period for each oil fraction. At 0% oil fraction (100% water fraction), 500 datasets were acquired. For any specific fraction of oil from 4 to 64%, 1500 datasets were acquired consisting of 500 datasets each measured for the three flow regimes.

In this highly dynamic and fluctuating environment with large datasets (24,500 datasets) from four incident angles across all measurements, the Feed Forward Neural Network employed to interrogate the lab data (e.g., Fig. 3a) converges very slowly and does not yield a prediction of sufficient accuracy (${R}^{2}$ score less than 0.95). In this case, it is more appropriate to use a Convolutional Neural Network fed with the complete transmission coefficient spectra from all measured angles as one set of training input data, instead of just one angle as tested in Fig. 3a and c. Unlike the complete color contour plots in Fig. 2c, here we only have four measurements angles limited by the antenna placement, but their locations around the perimeter of the PC provide symmetry information connected to the liquid distribution in the holes.

Similar to what was done in Fig. 2a for data tailoring, all the datasets are randomly split into two groups with the proportions 80:20 of non-overlapping training and test examples, respectively. For example, for a specific oil fraction and flow regime, there are 500 datasets with 400 sets randomly chosen as training input data and the remaining 100 used for testing. All training input data along with their labels (oil fraction and the specific flow regime) are then fed into a supervised 2-Dimensional Convolutional Neural Network (CNN) classifier to build a training model. The accuracy of the CNN model is then tested on the remaining 20% proportion of the test data. The 2D probability histograms in Fig. 4c and d show the prediction accuracy for flow regimes and phase fractions. The true flow regimes are verified by the wire-mesh sensor data and the side-glass camera imaging while the true fluid phase fractions are controlled and verified by sampling liquid in the loop. The overall prediction accuracy for flow regimes (Fig. 4c) is over 99%, and within one of the flow regimes—“Churn” flow, the overall prediction accuracy for oil fraction is 96% with a ${R}^{2}$ score of 0.998. Due to the churning and turbulent fluid dynamics along with some phase separation (Fig. 4b), “Churn” flow is the most difficult flow regime to characterize while the other two flow regimes, Stratified and Dispersed yield exemplary oil fraction prediction accuracy greater than 99%. It is worth noting that utilizing our configuration which includes a multiplexer, it takes less than 1 s to acquire a complete dataset with all angles and frequencies. Moreover, once the CNN model is trained offline it takes less than 10 milliseconds to predict the flow morphology and phase fraction. Therefore, this technology is certainly suitable for in-line real-time measurements.

In order to identify the limitations of this technology, in Fig. 4e we assess the predictive power of our method when the number of measurement frequencies is reduced while both training and testing the CNN model. The reduced number of frequencies are uniformly selected from the 128 discrete frequencies utilized to generate Fig. 4c and d. With a well-defined symmetry and homogenization, the prediction accuracies for stratified and dispersed flow regimes stay relatively high while the accuracy dropped to about 87% for “Churn” flow when 8 measurement frequencies were used. It is encouraging that, ${R}^{2}$ scores are all over 0.99 for all tests shown in Fig. 4e. This demonstrates the potential for robust prediction of phase fractions and flow regimes even with a limited amount of data consisting of 4 angles and 8 frequencies.

### Flow rate measurement with photonic crystal “Flow Conditioner” and differential pressure sensor

**Fig. 5:**
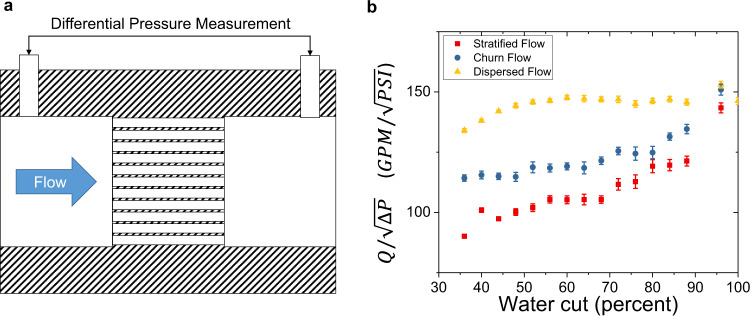
Pilot-scale experiments for flow rate measurements. **a** A longitudinal cross-section of this our integrated measurement device. The photonic crystal is in the center and the differential pressure gauge access the flow through two ports on either side of the photonic crystal. The blue arrow indicates the direction of the flow. **b** The rate-to-pressure coefficient, $A=Q/\sqrt{\triangle P}$, determined from the measured flow rate and pressure drop as a function of phase fractions (water cut in this case) for the three flow regimes tested in the field. Here *Q* is the volumetric flow rate with the unit of gallons per minute (GPM) and ∆*P* is the pressure drop across the PC structure with the unit of PSI. (Top) Yellow data points correspond to dispersed flow, (Middle) blue data points correspond to churn flow, and (Bottom) red data points correspond to stratified flow. The error bars represent one standard deviation of uncertainty for all data measured at the specific water cut and flow regimes.

In addition to a robust prediction of phase fraction and flow regime, we can exploit the hydrodynamic drag intrinsic to the photonic crystal in order to infer the flow rate by measuring the pressure drop across the structure. A longitudinal view of an exemplary integrated device is illustrated in Fig. 5a. A differential pressure measurement device is attached to two ports, one on the upstream and one on the downstream, side of the PC (see Methods). Similar to conventional Venturi meter theory^19^, the volumetric flow rate through the PC structure is given by the following equation: $Q=A\sqrt{\Delta P}$. Here *Q* is the volumetric flow rate and ∆P is the pressure drop across the PC structure. As for this specific rate-to-pressure coefficient *A*, for a conventional Venturi meter with a single orifice in non-turbulent single-phase flow at low-Reynolds number, *A* is a known function of density of the fluid as well as several parameters associated with the shape and geometries of the Venturi meter such as the beta ratio, throat area and the discharge factor^19^. However, for a multiphase flow that may exhibit laminar or turbulent regimes, *A* is a complicated function of phase fractions, flow regimes, density and viscosity of the individual phases, and the geometry/shape of the structure. While the density and viscosity of the individual phases may be determined through sampling, phase fractions and flow regimes of typical multiphase flows are highly dynamic. As a result, conventional flow rate technologies like, the Venturi meter, cannot reliably relate the differential pressure measurement to a flow rate measurement without one or more other devices to determine phase fractions and flow regimes^18–20,22,33–35^. Other flow meters, like the Coriolis meter, are susceptible to errors due to the propensity to entrain gas in the U-shaped tubes^21^.

In contrast, the PC does not suffer from these complications. We have an integrated piece of PC structure with electromagnetic and differential pressure measurements. The PC has a simple geometry consisting of bulk dielectric materials with an array of straight holes to minimize the potential for trapping phases. In addition, as shown in the field testing (Fig. 4), the technology provides highly accurate phase fraction and flow regime measurements that are necessary to determine *A*. Figure 5b demonstrates that given *Q* and Δ*P*, $A=Q/\sqrt{\Delta P}$, the rate-to-pressure coefficient, is a statistically unique value for the PC over the range of conditions tested in the field. Therefore, the determination of *A* can be integrated into the machine learning model for the oil fractions (water cut from 36 to 100% with a step size of 4%) and flow regimes we tested. In contrast, if the flow regime is unknown but the water cut is known, (e.g., 36% water cut), there is roughly a 50% variation in *A* (3 vertical points at 36% water cut in Fig. 5b) over the range of flow regimes tested; Alternatively, if the flow regime is known (e.g., dispersed flow regime), there is roughly 20% variation in determining the value of *A* (across the yellow data points in Fig. 5b). This demonstrates that PC technology yields significant improvement in measurement accuracy when both phase fraction (water cut in this case) and flow regime are determined from the microwave transmission measurement. For example after our CNN model predicts a 48% water cut and churn flow from a dataset, the variation in *A* has a small error bar on the order of 1% and we note a similar amount of error is applicable to the entire parameter space shown in Fig. 5b. With the knowledge of both phase fraction and flow regimes provided through our single integrated system, the pre-determined *A* value can be combined with a simple differential pressure measurements to predict volumetric flow rate with an error bar of less than 2% overall.

## Discussion

This work demonstrates that photonic crystals enable the characterization of multiphase flows by supporting the transmission of low energy (~1 mW) microwaves. In particular, the orientation specific transmission coefficient is sensitive to the spatial phase distribution in the crystal through the spatially varying contrast in dielectric constant^36^. These broadband measurements sample the entire pipe cross-section and are effectively coupled with Deep learning physics-based data analytics to infer phase fractions, and flow morphology in real time with better accuracy than existing commercial technologies. Moreover, we demonstrate that this approach is robust under the conditions and limitations imposed in an industrial setting. In particular we show that the measurement is applicable to dynamic flows, and the accuracy is not eroded by a significant reduction in data. In addition, when the phase fraction and flow rates are known we also demonstrate that the flow rate is accurately determined from the differential pressure necessary for the flow to pass through the photonic crystal. These finding should serve to significantly limit the cost associated with establishing these techniques in practice. For instance, we show that it is only necessary to sample 4 angles, and 8 microwave frequencies in order to achieve flow characterization of sufficient accuracy.

Moreover, unlike other intrusive sensing systems, our sensors and sensitive electronics are not in direct contact with the test fluid. Therefore, these components are not susceptible to corrosion and fouling. Alternatively, if the PC is eroded or fouled, we could simply replace the inexpensive PC “flow conditioner”. To test for corrosion or fouling, inject a slug of standard dielectric, then compare the associated measurement to an initial contour plot such as that shown in Fig. 1e or the leftmost panel of Fig. 2b. In addition, our PC based system can work across a wide range of water-cuts and potentially detect three-phase flow with a single set of measurements, without the limitations, such as specific fluid conditions, composition, and operational modes required by other intrusive methods^15,37–40^.

For field implementation, the pilot system (as shown in Supplementary Fig. 5, Fig. 4a and Fig. 5a) could be installed on a flowline. In some cases the process conditions (pressure, temperature, and chemical resistance ratings) may warrant the use of a modified system that requires flow diversion or enclosing the PC in a steel casing. In the latter scenario more compact or 2D RF/microwave antennas^41^ could be placed between the PC and the steel casing with microwave feedthroughs to transmit the microwave energy. Moreover the requisite microwave frequencies are similar to that of common Wi-Fi and 5 G standards, which means the hardware to generate/receive the microwaves is readily available. We anticipate that antenna designs may be improved by exploiting recent advances in electromagnetic metamaterials and metasurfaces^42–45^. Our technology could also be utilized to infer the composition of 3-phase flows due to the distinct dielectric properties of water, oil, and gas. It is certainly possible that the PC pattern and dielectric properties could be optimized for specific applications. Therefore, this work provides the basis for deriving novel and easy-to-deploy flow characterization techniques in support of process optimization.

## Methods

### Lab-scale equipment

In lab-scale experiments, we used a network analyzer (Agilent N5230A) to transmit electromagnetic waves in the microwave band (1–13.5 GHz) through the transmitting antenna at 1 mW power, and use the receiving antenna to measure the transmission through the structure. Both antennas are double ridge horns (Model 3115 from ETS-Lindgren). The polyethylene PC structure sits on top of a straight plastic rod made of PEEK (Polyether ether ketone) that is attached to a rotational stage (Zaber X-RST120AK). The PEEK rod is 60 cm in length and 2.5 cm in diameter and it is used to minimize electromagnetic interference between the stage motor and the microwave measurement hardware.

### Microwave transmission measurements

To generate the contour plots as shown in Fig. 1 and Fig. 2, we measure the transmission coefficient spectra (in decibles-milliwatt or dBm) as a function of frequency of incident waves at a specific angle/configuration (an example at zero incident angle is shown as Supplementary Fig. 1). By rotating the PC with the rotational stage, we can obtain a series of transmission coefficient spectra at any incident angle over the 360° range. The corresponding results can be represented as a color contour plot as shown in Fig. 1, Fig. 2, and Supplementary Fig. 2. In these plots the *x*-axis is the incident angle and the *y*-axis is the microwave frequency. The color in the contour plot represents the transmission coefficient at the specific frequency and incident angle. In general, red indicates higher transmission coefficient and blue indicates lower transmission coefficient. The color scale is shown on the right side of Fig. 1f. The transmission coefficient spectra contain a total of 201 frequencies between 1 and 13.5 GHz in Fig. 2b and Fig. 3a. Whereas, the plots in Fig. 2b and Fig. 3b, c, and d, consist of 33 frequencies over the same frequency band. The transmission coefficient spectra shown in Fig. 4 contain 401 frequencies from 4 to 26 GHz.

### COMSOL simulations

RF module in COMSOL is used to generate simulated microwave transmission contour plots for a total of over 1400 oil-filling configurations used in Fig. 2 and Fig. 3. The numerical experiments are parallelized by running 64 different oil-air configurations at the same time using 64 nodes on our research computing cluster. Each compute node here has 16 cores and our computing cluster has 504 compute nodes in total. The numerical experiments are performed in two-dimension with estimated microwave beam aperture, but otherwise utilize the same physical parameters as used in the lab experiments described in the main text (PC cross-section geometry and dielectric constants of all materials).

### Field testing

We used a different network analyzer (Agilent N5230A) to generate and analyze electromagnetic waves transmitted through the PC in the frequency band (4 GHz to 26 GHz). All 8 antennas are double ridged horns (Model 3116 C from ETS-Lindgren) connected to the network analyzer through two National Instrument RF multiplexers (Model 2597). Each multiplexer provides a connection between the source or receiver channel of the network analyzer and 4 of the antennas corresponding to the source or receiver antenna at each measurement angle. A complete measurement consists of iterating the collection of the transmission spectra over the 4 static measurement angles over a period of about 800 milliseconds (10-millisecond measurement time with network analyzer and 200 milliseconds to iterate multiplexer between the measurement angles). The integrated photonic crystal is made of PEEK (Polyether ether ketone) with relative permittivity of about *ε*_*r*_~2.9, a diameter of about 89 mm and a height of about 100 mm. The holes of the PC have a diameter of about 5.0 mm with a lattice constant of 6.67 mm. IsoparV^TM^ is manufactured by ExxonMobil and purchased through BrennTag. The differential pressure is measured through an OMEGA differential pressure transducer with model number 0305R732A11 and the pressure range from 0 to 250 inches of water pressure. The pressure measurement ports are located at a distance of about 20 cm from either face of the photonic crystal to provide for a stable pressure measurement. The research grade wire-mesh sensor is a customized probe purchased from HDZR Innovation consisting of a uniform 12 by 12 square grid (wire diameter of 0.3 mm) across a circular area with diameter of 89 mm (square grid window size of 6.85 mm by 6.85 mm), and we used the capacitive mode electronics (Model CAP200) during the two-phase water/oil field test. The wire-mesh sensor is placed 10 cm upstream of the PC (a distance of about 300 times the wire diameter), so it has minimal impact to the overall flow regime and phase fractions entering the PC^37,39,46–49^. As a result, the flow morphology and phase distribution inferred from this measurement is indicative of that entering the PC. An engineering drawing for this field prototype is shown in Supplementary Fig. 5. The side-glass camera is placed at the upstream side of this prototype. As a result, the camera is not affected by the disturbance of the wire-mesh sensor and more importantly the PC “flow conditioner”^31,32,48,50^, and its measurement (digital photos) should be indicative of what’s entering the wire-mesh sensor and the PC. The total volume fraction of gas in the flow loop is consistently below 2% during fluid filling or replacement, and pressurization up to 80 psi. So the impact of gas on the pilot test measurements is very small.

### Machine learning algorithms and associated statistics

Feed Forward Neural Networks are used to create Fig. 3. For predictions on experimental datasets (Fig. 3a), two dense layers are used with 80 and 40 hidden nodes with ‘tanh’ (hyperbolic tangent) as the activation function and Adam optimizer (learning rate is 0.005). For predictions on simulation datasets (Fig. 3c and d), two hidden-layers are used, each with 100 hidden nodes, with ‘tanh’ as the activation function and Adam optimizer. A Convolutional Neural Network (CNN) is used in Fig. 4, with two convolutional layers and a dense layer. The two convolutional layers have 64 and 32 feature maps, both with kernel size of 3 and stride of 2. The dense layer is used with 128 hidden nodes, exponential linear unit as the activation function and Adam optimizer (learning rate 0.00002).

For the training and testing with the field-test data, due to the lower signal to noise ratio at the higher microwave frequencies (our electronics are the predominant source of noise), we focused on the lower band of 128 frequencies over the total 401 frequencies in the range 4–26 GHz. We did not notice a significant difference when analyzing the data with 128 or 401 frequencies. The CNN model used in Fig. 4d with all training data in churn flow regime (6800 datasets, each dataset contain information from all angles and 128 frequencies) is trained in about 45 min with a single core of i5-8365U on a laptop. With a trained CNN model, the prediction of flow morphology and phase fraction with acquired dataset takes less than 10 milliseconds.

The 2D probability histograms used in Fig. 3 and Fig. 4 are very similar to a Confusion Matrix used in data science, with the exception that the probability of prediction is shown as pixel darkness instead of a counting number from the predicted result. The probability is normalized along each *y*-axis pixel to ensure that for each true oil fraction, the probabilities of predicted oil fractions sum to one.

The prediction accuracy is the probability of the predicted class being exactly the same as the true class, among all blind test data across all classes. Mathematically the prediction accuracy is the ratio of ${N}_{{{{{{{\mathrm{correct}}}}}}\; {{{{{\mathrm{test}}}}}}}}$ and ${N}_{{{{{{{\mathrm{all}}}}}}\; {{{{{\mathrm{test}}}}}}}}$, where ${N}_{{{{{{{\mathrm{all}}}}}}\; {{{{{\mathrm{test}}}}}}}}$ is the number of all blind test data and ${N}_{{{{{{{\mathrm{correct}}}}}}\; {{{{{\mathrm{test}}}}}}}}$ is the number of test data that are exactly predicted with the neural network model. Given this definition, it is clear the prediction accuracy does not account for the variance of incorrect predictions. Therefore, the prediction accuracy is a fairly harsh test of the accuracy of a model. The prediction accuracy is only applicable to discrete datasets. We note the prediction accuracy in the manuscript because it is regularly utilized in the data analytics community

In contrast, the ${R}^{2}=1-E/Y$ statistic quantifies the relative accuracy of a model through the ratio of the variability in the difference between the model and the data, or the residuals, *E*, and the variability in the data, *Y*^51^. When ${R}^{2}=1$, $E=0$, and the model accounts for all of the observed variability in the data. When considering experimental data, measurement error in the form of random electrical noise or errors associated with liquid measurement techniques ensure $E > 0$ and ${R}^{2} < 1$. With a small number of classes (large step size for phase fraction) such as in Figs. 3a, 4d, and 4e, the ${R}^{2}$ score and prediction accuracy are similar (all over 0.99) because the random error is smaller than the step size. We are only able to test a larger number of classes (small step size for phase fraction) numerically. In this case there are no measurement errors, and the statistics are a true test of the ability of the model to account for the variation in the true class. As shown in Fig. 3c, the ${R}^{2}=0.990$ score accurately quantifies the variability of the model prediction which is predominantly limited to the light gray ± 1 px (1.3%) adjacent to the 1-1 line. As a result of the increase in variability of the model prediction the prediction accuracy is significantly less than *R*^*2*^. However, the high *R*^*2*^ value indicates the high accuracy (~1%) of the model across the range of true phase fractions.

Uncertainty is a key metric to compare our technology to those that are already commercially available, and it can be estimated with the above mentioned statistics along with our data. To comply with ISO3170, uncertainty is defined as a percentage range with 95% confidence of prediction (approximately twice the standard deviation). For Fig. 4d with dynamic churn flow, each bin/pixel represents 4% of phase fraction with a prediction accuracy or confidence of 96% and as a result we can estimate that the uncertainty for this case is about ±2% (similar to the size of the bin). Similarly, we can estimate that the uncertainty is about 1.3% for the simulation data in Fig. 3c.

## Supplementary information

Supplementary Information

## Data Availability

The raw data are not publicly available due to company policy. The raw data that support the findings of this study are available upon reasonable request from the corresponding authors L.F. and J.J.V., subject to approval from ExxonMobil.

## Code availability

The codes are not publicly available due to company policy. The codes that support the findings of this study are available upon reasonable request from the corresponding authors L.F. and J.J.V., subject to approval from ExxonMobil.
