# Supplementary material for: Multiphase flow detection with photonic crystals and deep learning
Source: Nat Commun. 2022 Jan 28;13:567. doi: 10.1038/s41467-022-28174-2 (PMC8799677; doi:10.1038/s41467-022-28174-2)
Supplement: Supplementary file 1 — Supplementary Information [file 41467_2022_28174_MOESM1_ESM.pdf]

Supplementary Information:

Multiphase Flow Detection with Photonic Crystals and Deep Learning

Lang Feng[*1], Stefan Natu[1§], Victoria Som de Cerff Edmonds[2], John J.Valenza[*1]

[1]Corporate Strategic Research, ExxonMobil Research and Engineering, 1545 Route 22 East, Annandale, NJ, 08801, USA; [2]Research and Engineering IT, ExxonMobil Technical Computing Company, 1545 Route 22 East, Annandale, NJ, 08801, USA; § Current Address: Amazon Alexa, 7 W 34th St, New York, NY, 10001, USA; * *E-mails:* Lang Feng lang.feng@exxonmobil.com; *John Valenza john.j.valenza@exxonmobil.com

Contents:

Supplementary Figure 1

Microwave transmission coefficient spectra at zero incident angle

Supplementary Figure 2

Microwave transmission contour plot from 0 to 360 degrees

Supplementary Figure 3

Microwave transmission polar intensity plot

Supplementary Figure 4

Evolution of the polar intensity plot as the oil filling fraction increases from 0% to 100%

Supplementary Figure 5

Engineering drawing for the field test prototype

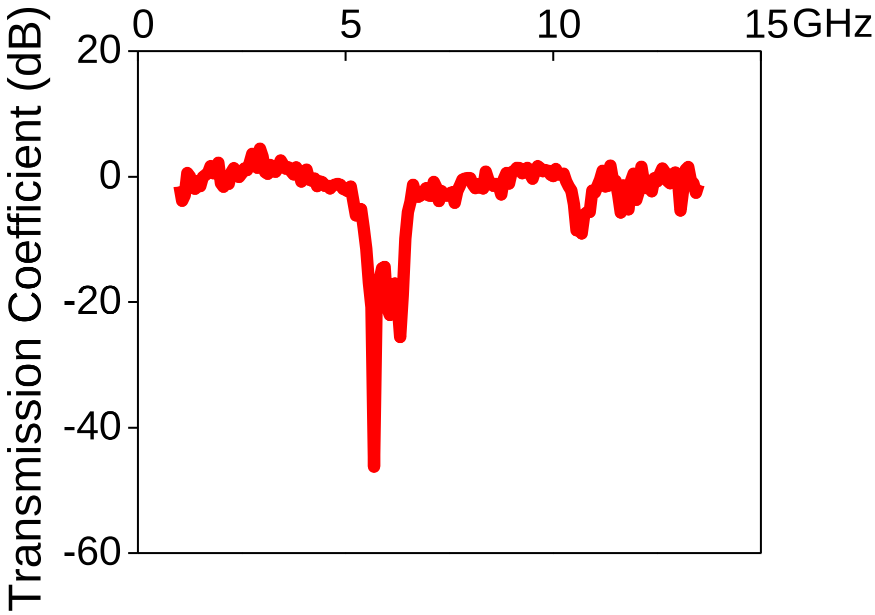

Supplementary Figure 1 | Microwave transmission coefficient spectra at zero incident angle

An example of transmission coefficient spectra (in decibles-milliwatt or dBm) as a function of frequency of incident waves at zero incident angle (as in Figure 1(a)).

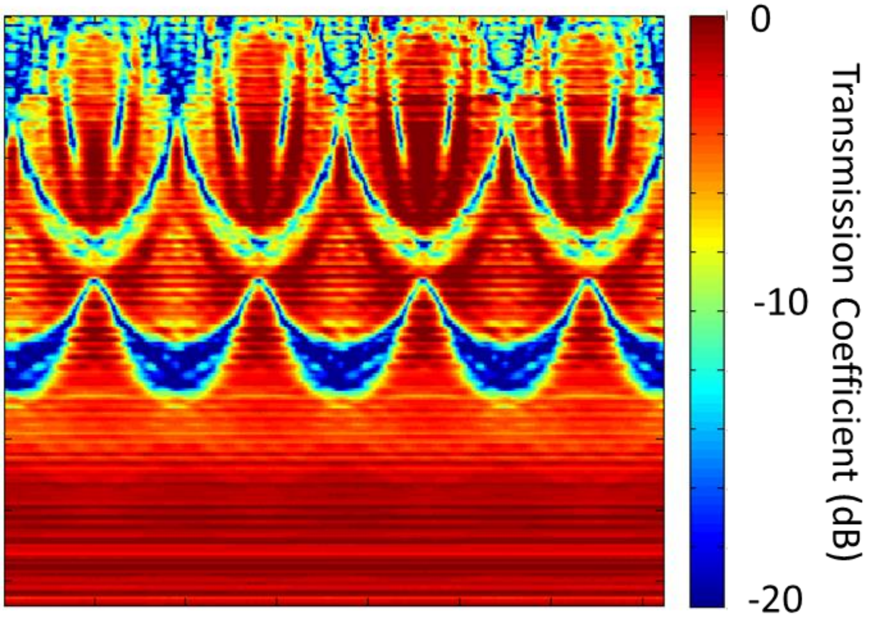

Supplementary Figure 2 | Microwave transmission contour plot from 0 to 360 degrees

An example of the measured microwave transmission contour plot from 0 to 360 degrees for the 0% oil filling fraction. The contour plot contains 360 equally spaced angles in the range of 0 to 360 degrees, and 200 equally spaced frequencies in the range 1.0 GHz to 13.5 GHz, with a well-defined symmetry due to the near-perfect photonic band structure (e.g. as compared to Figure 1(d) X-M).

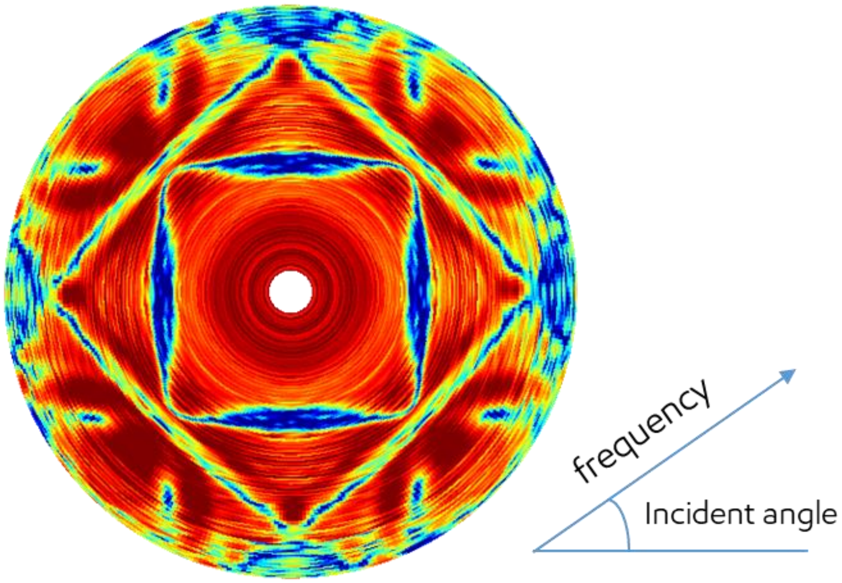

Supplementary Figure 3 | Microwave transmission polar intensity plot

An example of the measured microwave transmission contour plot as a polar intensity plot from 0 to 360 degrees for the 0% oil filling fraction. Here, the angular axis is the orientation or rotational angle and the radial axis is the normalized frequency. Both Supplementary Figures 2 and 3 show a 4-fold symmetry that is a result of the symmetry of the square lattice of holes. In addition the square-shaped blue zones in Supplementary Figure 3 represent the first and second Brillouin zones of the structure. The microwave transmission coefficient color scale is the same as that shown in Supplementary Figure 2.

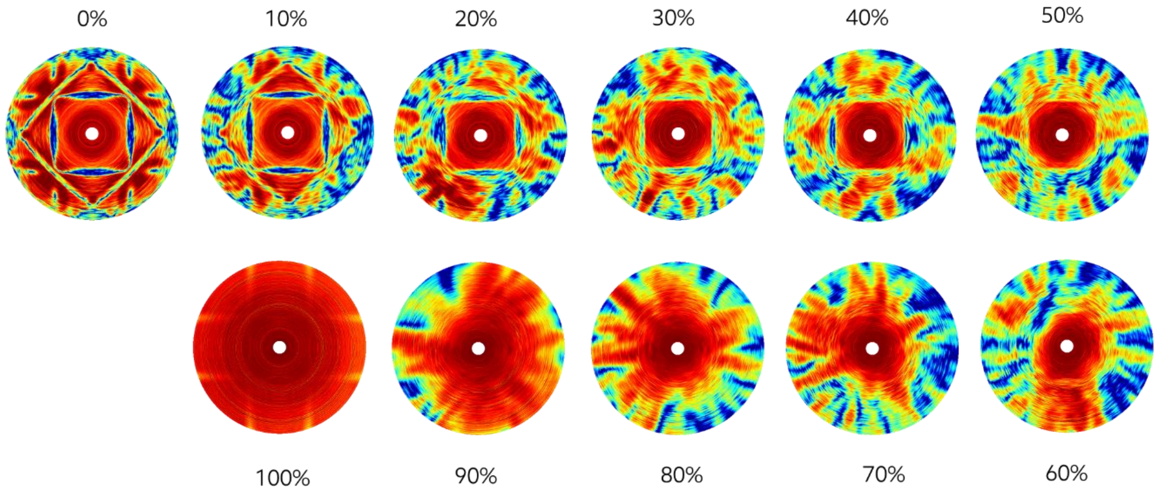

Supplementary Figure 4 | Evolutoin of the polar intensity plot as the oil filling fraction increases from 0% to 100%

Examples of the measured microwave transmission contour plots as a polar intensity plot from 0 to 360 degrees from 0% to 100 % oil filling fractions (with a step size of 10%). Similar to Supplementary Figure 3, the angular axis is the orientation or rotational angle and the radial axis is the normalized frequency. The microwave transmission coefficient color scale is the same as that shown in Supplementary Figure 2.

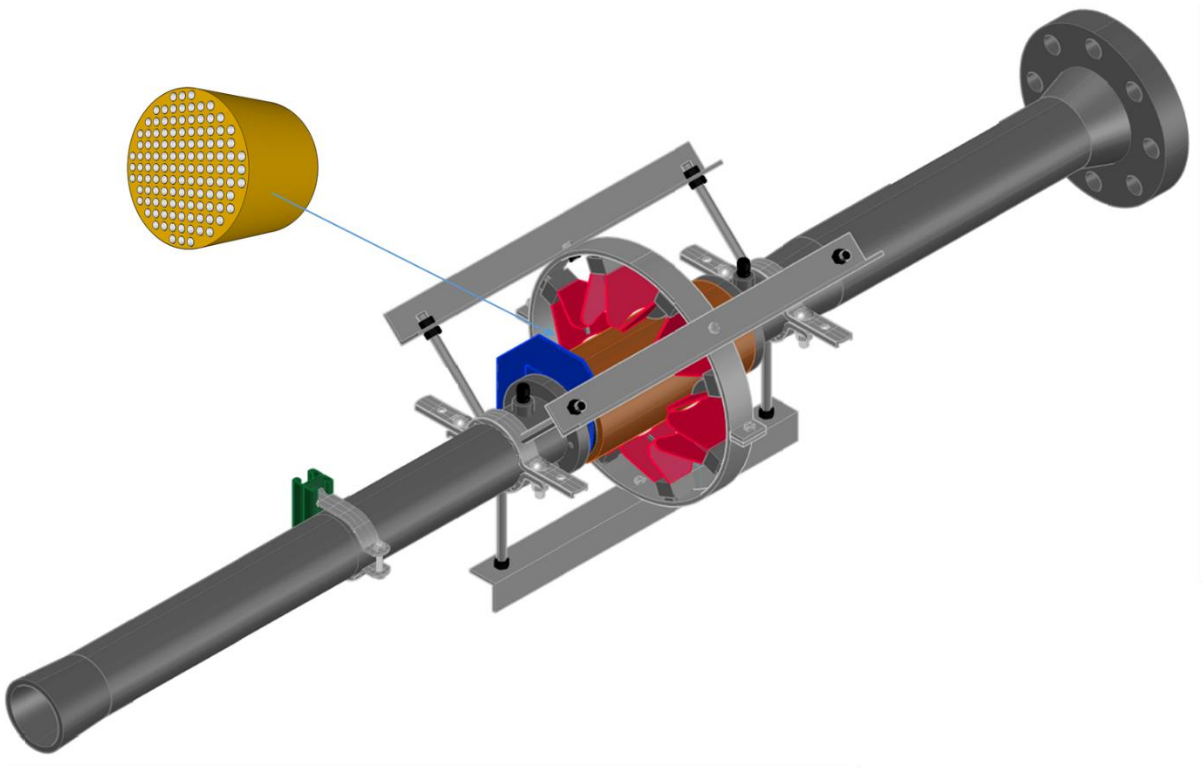

Supplementary Figure 5 | Engineering drawing for the field test prototype.

In our field test prototype, 8 antennas (Red - Model 3116C double ridged horn from ETS-Lindgren) are connected to the network analyzer through two National Instrument RF multiplexers (Model 2597). The integrated photonic crystal (Brown) is made of PEEK (Polyether ether ketone) with a diameter of about 89 mm and a height of about 100 mm. The holes of the PC have a diameter of about 5.0 mm with a lattice constant of 6.67 mm. The differential pressure is measured through an OMEGA differential pressure transducer with model number 0305R732A11 and the pressure range from 0 to 250 inches of water pressure. The pressure measurement ports are located at a distance of about 20cm from either face of the photonic crystal to provide for a stable pressure measurement. The research grade wire-mesh sensor (Blue) is a customized probe purchased from HDZR Innovation with 12 by 12 grids of spatial resolution across a circular area with diameter of 89 mm, and we used the capacitive mode electronics (Model CAP200) during the two-phase water/oil field test.
